# Successful Application of the Gamma-Interferon Assay in a Bovine Tuberculosis Eradication Program: The French Bullfighting Herd Experience

**DOI:** 10.3389/fvets.2018.00027

**Published:** 2018-02-27

**Authors:** Nicolas Keck, Maria-Laura Boschiroli, Florence Smyej, Valérie Vogler, Jean-Louis Moyen, Stéphanie Desvaux

**Affiliations:** ^1^Laboratoire Départemental Vétérinaire de l’Hérault, Montpellier, France; ^2^University Paris-Est, French Reference Laboratory for Tuberculosis, French Agency for Food, Environmental and Occupational Health & Safety (Anses), Maisons-Alfort, France; ^3^Direction Départementale de la Protection des Populations, Nîmes, France; ^4^Service Régional de l’Alimentation, Direction Régionale de l’Alimentation, de l’Agriculture et de l’Occitanie, Montpellier, France; ^5^Laboratoire d’Analyses et de Recherche de Dordogne, Coulounieix-Chamiers, France; ^6^Unité Sanitaire de la Faune, Office National de la Chasse et de la Faune Sauvage (ONCFS), Birieux, France

**Keywords:** bovine tuberculosis, screening, gamma-interferon assay, field performances, strategic use, eradication

## Abstract

In the French Camargue region, where bovine tuberculosis had been enzootic for several years in bullfighting cattle herds, the gamma-interferon (IFN) assay was used since 2003 in parallel with the intradermal test in order to increase overall disease detection sensitivity in infected herds. This study presents the results of a field-evaluation of the assay during a 10-year period (2004–2014) of disease control and surveillance program and explores the particular pattern of IFN assay results in bullfight herds in comparison to cattle from other regions of France. The low sensitivity [59.2% (50.6; 67.3)] of IFN assay using the tuberculin stimulation could be related to the poor gamma-IFN production from bullfight cattle blood cells which is significantly lower than in animals of conventional breeds. The characteristics of the assay were progressively adapted to the epidemiological situation and the desired strategic applications. Data analysis with a receiver operating characteristic curve based on a simple S/P value algorithm allowed for the determination of a new cutoff adapted for a global screening, giving a high specificity of 99.9% results and a high accuracy of the assay. Having regularly risen to above 5% since 2005, with a peak around 10% in 2010, the annual incidence dropped to under 1% in 2014. The positive predictive value relative to the bacteriological confirmation evolved during the years, from 33% in 2009 to 12% during the last screening period, a normal trend in a context of decreasing prevalence. The estimated rate of false-positive reactions during screening campaigns was 0.67%, confirming the high specificity of the test, measured in bTB negative herds, in this epidemiological context. The proportion of false-positive reactions decreased with the age and was higher in males than in females. Although these results indicate that the IFN assay is accurate in the field, it also emphasizes great differences between interferon quantities produced by bullfight cattle blood samples compared to those of classical bovine breeds, which underlines the necessity to adapt the algorithms and combinations of the assay according to local epidemiological contexts.

## Introduction

Bovine tuberculosis (bTB) is a zoonotic disease mainly caused by infection with *Mycobacterium bovis*. Although it is still a major public health problem in some countries, it is more regarded nowadays as an economic problem for agriculture in developed regions of the world where schemes for tuberculosis control and eradication have considerably reduced the prevalence of the disease and sometimes to achieve disease-free status (1). However, these efforts must be maintained, since some countries encounter persistence or reemergence of *M. bovis* infection in their cattle populations (2).

The reasons for these failures are complex and partly due to the difficulties of *ante-mortem* diagnosis of tuberculosis in cattle which remains extremely challenging. In most countries, eradication programmes are based on regular testing and removal strategy by compulsory slaughter of reactors, using the intradermal tuberculin tests with tuberculin purified protein derivative (PPD) to detect infected animals (3). Although this tool has historically been useful in reducing the incidence of tuberculosis, it has shown a lack of sensitivity (4, 5). Furthermore, the skin test is sometimes difficult to use in the field, which may entail operator errors and false-negative results. Limitations in the specificity due to non-specific hypersensitivity reactions in cattle infected with non-pathogenic environmental mycobacterial species are also observed (4). As a consequence, alternative tests were developed to enhance the success rate of infection diagnosis. Particularly, the gamma-interferon (IFN) assay was evaluated and adopted as an official test in several countries (6).

In France, the national tuberculosis surveillance and control program in place since the 1950s and using intradermal tuberculin test has contributed to the drastic reduction of bTB in the country which is recognized as officially “bTB free” since 2001. However, in Camargue, south of France, a region of marshlands where cattle breeding consists mostly of bullfight herds, bTB has been enzootic for several years, with an average annual incidence of 5.5% from 1996 to 2005. Control of the disease has been hampered by the lack of sensitivity of skin test in this population, the free range status of the herds and the breeding techniques which favor contact between animals of different herds. Consequently, the very high level of infection was probably underestimated and 80% of bTB cases were discovered at the slaughterhouse (7). As a consequence of this serious situation, local authorities decided to implement a strengthened bTB control program based on several measures including the use of IFN assay. The decision to use this assay was taken to improve early detection of infection at the farm level (6, 8). In the absence of national or international guidelines for gamma-IFN assay use and interpretation, regional laboratories in collaboration with the National Reference Laboratory had to evaluate and regularly adapt the context of use as well as the interpretation scheme in the context of an ISO 17025 quality assurance system and the regulatory framework for validation of reagents employed for screening notifiable diseases. This study presents the results of the IFN assay field-evaluation during a 10-year period (2004–2014) of disease control and surveillance program and explores the particular pattern of this assay in bullfight herds populations compared to other kind of cattle breeds. The surveillance outcome for the Camargue region is also presented and the strategic use of ancillary tests for the control of bTB is discussed.

## Materials and Methods

### Animal Population Characteristics and Ethics Statement

There are around 250 bullfighting cattle herds in the Camargue region (around 30,000 animals of 2 main breeds, “raço di biou” and “brave”). Animals are either dedicated to bullfighting or to local traditional games (in arena or in villages) and are bred in very extensive conditions. As they are mostly used for entertainment or for reproduction, animals are kept for long time periods within the herd and it is quite common to find animals older than 10 years (up to 11% animals in the herd).

This bovine population is very rarely moved out from Camargue region and has almost no contact with other cattle operations. This explains why bTB is confined to this specific population and does not affect other production systems in the region.

The domestic animals used in this study met the definition of “farm animals”, which are not currently covered by French regulations (Décret 2013-118 dated the February 1, 2013, from the French Ministry of Agriculture). The owners of the animals were informed of tests performed on their animals, since all samples were collected during compulsory sanitary investigations.

### Field Data

The specificity (Sp) of IFN assay was evaluated in a population of 1,008 animals aged more than 2 years, sampled from six herds considered as bTB free according to the following criteria: (i) no cattle with suspect lesions at the slaughterhouse had been confirmed *M. bovis* culture positive within the last 10 years; (ii) no animals had tested positive to single intradermal cervical skin test (SICT) during the last 6 years (annual screening); and (iii) no epidemiological link with infected herds had been established during the last 6 years. Herds were distributed homogeneously in the Camargue area.

The sensitivity (Se) was evaluated with a data set made of 142 infected animals sampled for IFN assay in 18 depopulated herds from 2006 to 2010.

Field performance of the assay and results of quality controls were also evaluated using data from two global screening programs performed in 2009–2011 (14,199 animals) and 2012–2014 (17,534 animals) in all herds (more than 2 years old animals). The association between a false-positive gamma-IFN response and certain individual characteristics (age, gender, breed) was evaluated using data from herds with bTB-free status in the 2012–2014 global screening period from which individual information was available (11,931 out of 15,532 cattle from bTB-free herds).

The optical density (OD) values obtained with the IFN assay in Camargue were compared to those obtained from 44 infected animals among 24 different herds [comparison of bovine purified protein derivative (PPDB) OD values] and 13,474 animals (comparison of mitogen OD values) from “conventional” cattle from Dordogne (South west France) sampled from 2009 to 2011.

For each infected animal, the macroscopic visible lesions (VLs) were characterized and classified according to the following scoring:

- Level 1: lesions confined to one or several lymph nodes of a same anatomic region.
- Level 2: lesions in an organ with or without lesion of one or more lymph nodes of the same anatomic region, or any caseous lesion of at least one lymph node.
- Level 3: lesions on at least two different anatomic regions.

### bTB Case Definition

Herds were considered infected when at least one animal presented one or more of the following criteria for being considered infected:

- observation of clinical signs of bovine tuberculosis associated with a SICT positive result,
- isolation of *M. bovis* by culture,
- association of a positive SICT or IFN assay result with histopathology positive bTB lesions,
- association of a positive PCR result with histopathology positive bTB lesions or a SICT or IFN assay positive result,
- observation of bTB VL in an animal from a previously demonstrated infected herd.

### Animals Testing

Blood samples were collected in heparinized tubes the same day SICT was performed, transported at ambient temperature (22 ± 5°C, avoiding extreme temperatures) to the laboratories and processed within 8 h postcollection. Stimulation of whole blood was done with PPDB and avian purified protein derivative (PPDA) (Prionics AG, Schlieren, Switzerland). PPDB and PPDA were prediluted with phosphate buffer saline (PBS) to achieve a final assay concentration of 20 µg/ml. Pokeweed mitogen (PWM) at 5 µg/ml and NIL antigen PBS were used, respectively, to control the blood cells ability to produce gamma-IFN and to detect a non-specific gamma-IFN production. Whole blood cultures were performed with 24-well plates from 2006 to 2009 (1.5 ml of heparinized blood with 100 µl of antigen solution) and 96-well plates from 2009 to 2012 (250 µl of blood with 25 µl of antigen solution, 2 stimulation wells per antigen). Blood samples were evenly mixed before aliquoting. Plates were incubated at 37°C in a humidified atmosphere for 16–24 h, then centrifuged at 500 *g* for 10 min at room temperature. After incubation, approximately 100 µl (96-well plates) or 500 µl (24-well plates) of plasma were removed from above the sedimented red cells using a variable-volume pipette. The absence of significant effect of vessel geometry has been demonstrated by Schiller et al. (9). Plasma samples were tested using Bovigam^®^ (Prionics AG, Schlieren, Switzerland) in duplicate wells. The same analytical protocol was used in Camargue and Dordogne regions. Some animals were tested with the SICT using the official bovine PPD (Synbiotics, France).

PCR and mycobacterial culture were performed on tracheobronchial, retropharyngeal, and mediastinal lymph nodes presenting or not VL at the slaughter house. Lymph nodes were analyzed individually. Culture was performed according to the French *ad hoc* guideline, using solid Lowenstein and Coletsos agar after decontamination with 4% H2SO4 solution neutralized by adding a 6% NaOH solution. After mechanical lysis of tissue, DNA was extracted by using the QIAamp DNA mini kit (Qiagen) or by Magvet MV384 (Life Technologies) with a King Fisher KF96 automate, following the manufacturer’s instructions. PCR was performed with a commercial kit (LSI VetMAXTM *Mycobacterium tuberculosis* Complex PCR Kit 2 wells) targeting IS*6110*, which is present in all species of the *M. tuberculosis* complex (10): 5 μl of the extracted DNA was mixed with 20 µl of reaction mix and the reaction was carried out at 50°C for 2 min (1 cycle), followed by one cycle of 10 min at 95°C and 40 cycles of 15 s at 95°C and 1 min at 60°C. Results were interpreted following the manufacturer’s recommendations and by comparison with negative and positive controls. Thermolysates of bacterial isolates were confirmed as *M. bovis* by Luminex spoligotyping as described by Zhang et al. (11).

### Data Analysis

The performance of the IFN assay was determined for the following positive cutoff recommended for the Bovigam^®^ kit, designated as the historical positive cutoff: (mean OD PPDB − mean OD NIL ≥ 0.1) and (mean OD PPDB − mean OD PPDA ≥ 0.1). Results were excluded when the OD value was higher than 0.3 for the NIL antigen-free sample (12) or less than 0.5 for the PWM-treated sample (9).

The Se and Sp were evaluated with their 95% exact binomial confidence interval (CI) at the individual level but also at the herd level, using the following formulas:

- Se herd level = 1 − (1 − Se ind)*^n^* considering an average number of *n* = 3 infected animal per herd (based on field data).
- Sp herd level = (Sp ind)*^n^* considering an average number of *n* = 76 animals per herd.

Additionally for all samples, the S/P value was calculated as proposed by Faye et al. (13): (mean OD PPDB − mean OD PPDA)/(mean OD PC − mean OD NC) where PC is the mean OD value of the positive control of the ELISA plate and NC is the mean OD value of the negative controls. The dilution of the positive control was adapted for each batch of ELISA kit to ensure a constant OD value of the positive control for all batches.

These data were used to build a receiver operating characteristic (ROC) curve to examine the impact of different cutoff values on the individual sensitivity and specificity relative to bacteriology and to define an optimal diagnostic cutoff value ensuring a high specificity adapted for global screening. ROC curves were performed using roctab command in Stata (non-parametric ROC analysis). Cutoff values evolved over time in order to be optimized and adapted to the epidemiological situation and the bTb control scheme (Table 1).

**Table 1:** Different cutoff values used over time during the eradication program.

Years	Epidemiological context	Objectives	Cutoff	Algorithm applied	Quality controls
2003–2008	High prevalence	Improve farm level detection and detect new tuberculosis cases	“Historical” positive cutoff	(OD PPDB − OD NIL ≥ 0.1)And(OD PPDB − OD PPDA ≥ 0.1)	OD NIL < 0.3

2009–2010	High prevalence	Maximize the sensitivity of the global population screening	Positive cutoff	(OD PPDB − OD PPDA)/(OD PC − OD NC) ≥ 0.04And(OD PPDB − OD NIL)/(OD PC − OD NC) ≥ 0.04	OD NIL < 0.3OD PWM > 0.4

			Suspect cutoff	(OD PPDB − OD NIL)/(OD PC − OD NC) ≥ 0And0.02 ≤ (OD PPDB − OD PPDA)/(OD PC − OD NC) ≤ 0.04Or(OD PPDB − OD PPDA)/(OD PC − OD NC) ≥ 0And0.02 ≤ (OD PPDB − OD NIL)/(OD PC − OD NC) ≤ 0.04	

2012–2014	Decrease of incidence	Improve the specificity of the global population screening	Positive cutoff	(OD PPDB − OD PPDA)/(OD PC − OD NC) ≥ 0.04	OD NIL/(OD PC − OD NC) < 0.125OD PWM/(OD PC − OD NC) > 0.125

The OD values from infected Camargue cattle were compared to those obtained from “conventional” cattle using a two-sample *T*-test with unequal variances (test command in Stata).

Logistic regression models to assess the association between a false-positive gamma-IFN response and certain individual characteristics (age, gender, breed) were built on data from 2012 to 2014 global screening programs (logistic command adjusting for herd clustering effect in Stata). A false-positive gamma-IFN response was assumed on all IFN positive results from bTB-free herds which were not confirmed by bacteriology and/or PCR.

## Results

### Performance of the Assay

#### Characteristics of the Assay

Results for the IFN assay field performance evaluation using historical cutoff values are presented in Table 2. The low sensitivity at the individual level was compensated at the herd level considering the high bTB within-herd prevalence during the first years of IFN assay use (average number of three infected animals per herd). The very high specificity at individual level was slightly hampered at herd level but remained above 90% despite the high average number of animals in bullfight herds.

**Table 2:** Results for the IFN assay field performance evaluation using “historical” cutoff.

Test result	Value% (95% CI)
Se—individual level (*n* = 142)	59.2 (50.6; 67.3)
Se—herd level^a^	93.2 (88.0; 95.5)
Sp—individual level (*n* = 1,008)	99.9 (99.4–100)
Sp—herd level^b^	92.7 (63.3; 100)

*^a^Assuming three infected animals per herd*.

*^b^Assuming a mean herd size of 76 animals*.

The relation between (OD PPDB − OD PPDA) and the level of observed lesions of infected animals was assessed using analysis of variance. No significant differences were observed (Table 3).

**Table 3:** Mean (OD PPDB − OD PPDA) values according to the level of visible lesion.

Level of visible lesions	*N*	Mean (OD PPDB − OD PPDA)
Level 1	38	0.1499
Level 2	47	0.1743
Level 3	46	0.1333

#### Comparison of Gamma-IFN Production between Bullfight and Conventional Cattle Breeds

**Figure 1:**
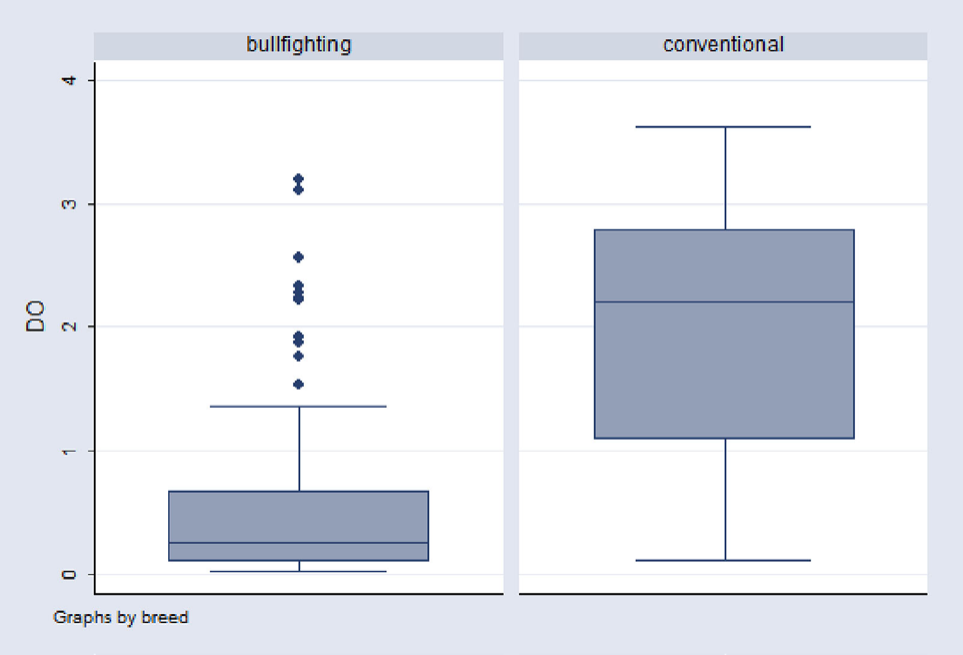
Distribution of optical density (OD) values for PPDB antigen in two infected populations: bullfighting (*N* = 142) and conventional cattle (*N* = 44). The upper lines in the boxes represent the 75th percentile (P75), the middle line represents the median (P50), and the lower line in the box represents the 25th percentile (P25). The ends of the whiskers represent minimum and maximum OD values.

Figure 1 shows that gamma-IFN production in stimulated whole blood from infected bullfighting cattle was much lower than in infected conventional cattle. Indeed, the average OD value for gamma-IFN produced in response to PPDB antigen in the population of Camargue bullfight cattle (Mean OD = 0.489, *n* = 142) was significantly different (*p* < 0.0001) from the average OD value in conventional cattle (Mean OD = 1.943, *n* = 44). The average PPDB OD value for infected bullfight cattle appeared very near the historical cutoff value. This could explain the low individual sensitivity using that cutoff and suggests that using a test with a better detectability could increase the sensitivity without degrading specificity.

In the whole population of bullfight cattle sampled from 2009 to 2011 (*n* = 14,199), the mean OD value obtained in wells stimulated with mitogen was 1.123 (1,091–1,155) while results obtained for cattle from the Dordogne region during the same period (*n* = 13,474) showed a significantly higher mean OD value of 2.3 (2.287–2.319). These results indicate that the criteria for discarding samples with limited gamma-IFN production according to the mitogen’s OD value should be determined according to the characteristics of the studied animal population.

### Assay Optimization and Definition of New Parameters

#### Cutoff Determination

**Figure 2:**
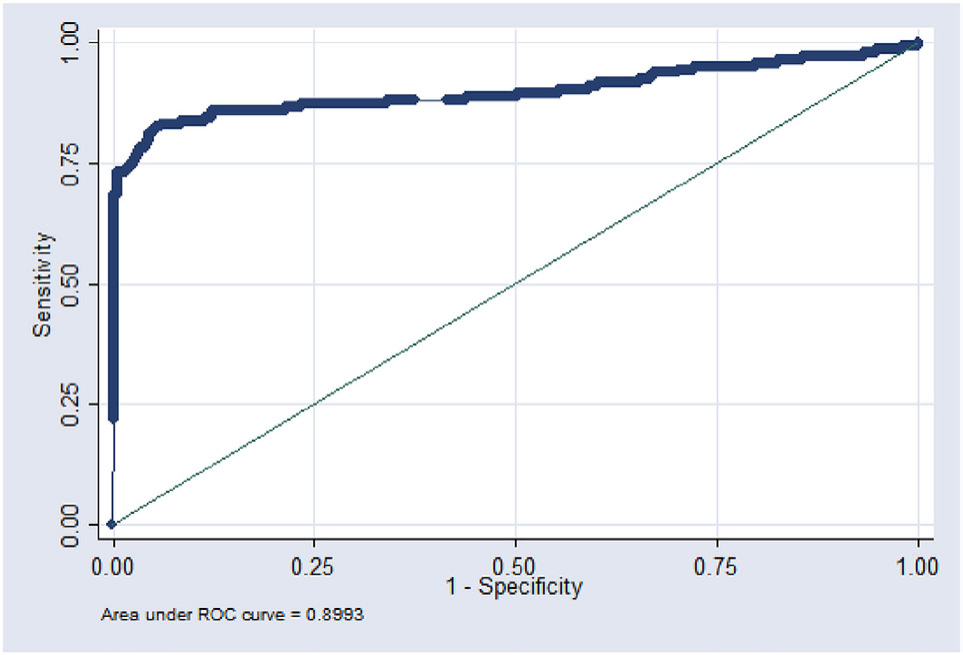
Receiver operating characteristic (ROC) curve established by using the S/P quantitative value.

Figure 2 presents the ROC curve using the S/P quantitative value, indicating an area under the curve (AUC) close to 0.9 giving indication of a high accuracy of the IFN assay taking into account previously published guidelines (14). The new S/P cutoff, (mean OD PPDB − mean OD PPDA)/(mean OD PC − mean OD NC) = 0.04, presented a high specificity adapted for global screening (Table 4). Furthermore this cutoff using a unique value was easier to evaluate than the historical one. As recommended for interlaboratory standardization (15), interpretation of the S/P value enabled to take into account the variability of raw OD values, which are absolute measurements influenced by test parameters and photometric instrumentation.

**Table 4:** Characteristics of the IFN assay for various cutoff values according to ROC simulation.

	Se (95% CI)	Sp (95% CI)
Cutoff S/P = 0.03	64.1 (55.61–71.96)	99.8 (99.29–99.98)
Cutoff S/P = 0.04	59.15 (50.6–67.32)	99.9 (99.45–100)
Cutoff S/P = 0.05	56.34 (47.77–64.64)	99.9 (99.45–100)

#### Quality Control Parameters

The mean OD for NIL antigen was 0.073 (0.071–0.075, *n* = 14,209) with 97.7% of the values below 0.3 confirming this value as an appropriate cutoff above which the result should be considered uninterpretable. Indeed, we observed that a high OD value obtained in wells stimulated with NIL antigen could entail false-negative results (unpublished results). The criteria previously described by Coad et al. (12) for controlling non-specific reactions was definitively adopted (OD NIL < 0.3 for valid result).

On the other hand, the criteria for controlling the immunocompetence of the blood cells (OD PWM) was adapted to take into account the low reactivity of this local bovine population. Thus, a value of 0.3 was adopted instead of the initial 0.5 cutoff.

With the introduction of these controls and adaptations, a result was classified as valid if OD NIL < 0.3 and OD PWM > 0.3. From 2012, those cutoff were converted to take into account the control values and samples were excluded when OD NIL/(OD PC − OD NC) > 0.125 or OD PWM/(OD PC − OD NC) < 0.125.

### Strategic Use and Field Performances for bTB Control

#### Context of Use and Evolution of the Incidence Rate

The IFN assay was first used in a limited number of herds and then, between 2006 and 2008, it was applied throughout the region for a screening program in farms considered at risk of being infected (i.e., epidemiologically linked to an outbreak, sanitation program during the previous 5 years, farms in which animals were never or rarely sent to slaughter). In 2008, the use of IFN assay was imposed for premovement controls. From 2009, it was decided to organize a general screening of the Camargue bullfight population. The objective of this first general screening was to maximize the sensitivity to detect as many cases as possible, thus a suspect cutoff was set-up (algorithm shown in Table 1). During the second screening program in 2012–2014, this suspect cutoff was eliminated in order to achieve greater specificity. Global screening was systematically organized over two years, starting in September and ending in June. During these periods, half of the farms was screened with IFN assay (cattle over 24 months) each year, while the other half was screened with the skin test (cattle over 12 months). Other actions were also implemented, particularly those concerning the reception conditions of animals in the arenas for reducing risky practices, but also through information and awareness of farmers and veterinarians.

**Figure 3:**
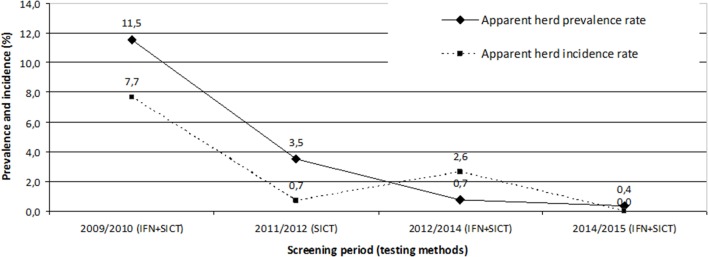
Evolution of the apparent prevalence and incidence rates during the two global screening periods. During these periods, half of the farms was screened with IFN test (cattle over 24 months) each year, while the other half was screened with the skin test (cattle over 12 months), except during the 2011/2012 period when all farms were screened only with the skin test.

The annual apparent incidence rate increased gradually from 2004 to 2008 (from 3 to 6%) and most of the outbreaks were detected during this period (68 outbreaks detected from 2004 to 2008 vs. 45 in 2009 to 2014). Since the first global screening period (2009–2011), the annual incidence rate shifted from 7% in 2010 to 0.7% in 2014 with only two new outbreaks detected in the field and no detection at the slaughterhouse (Figure 3).

#### Evolution of the Positive Predictive Value

As shown in Table 5, the positive predictive value (PPV) of a positive IFN assay relative to the bacteriological confirmation declined significantly between the first (near 20%) and the second screening period (12.5%). This is a normal trend in a context of decreasing prevalence. The number of non-tuberculous mycobacteria cultured from samples of reactor animals increased since 2010, corresponding to this screening pressure and the decrease of PPV (unpublished results). The SICT suspect rate seemed very low which may be linked with the particularly low performances obtained with this test in this specific herd production system.

**Table 5:** Results of the two global screening campaigns.

Screening period	Herds tested with SICT	Herds with positive SICT result (%)	Herds tested with IFN assay	Animals sampled^a^ for IFN assay (% samples with valid results)	Not valid samples due to NIL criteria	Not valid samples evaluated due to PWM criteria	Herds with positive IFN assay (%)	Herds with positive or doubtful IFN assay (%)	IFN assay positive animals (%)	PPV of IFN assay
2009–2010	232	2 (0.86)	204	14,199 (81.8)	329	2,247	55 (27.0)	102 (50.0)	102 (0.7)	19.6%
2012–2014	274	4 (1.5)	244	17,534 (90.3)	Not available	Not available	58 (23.8)	–	120 (0.7)	12.5%

*^a^Only bovines aged more than 24 months were sampled for IFN testing*.

#### Evolution of the Specificity Value

The rate of apparent false-positive reactions on bTB-free herds estimated for the 2012–2014 campaign was 0.69% (108/15,532) corresponding to a specificity of 99.31%, thus slightly above the expected rate initially estimated. The available variables that may help to explain the frequency of false-positive reactions were age, gender and breed. In univariable logistic regression analysis, these three variables influenced the occurrence of a positive IFN assay result while in multivariable analysis, only age and gender effects were confirmed (the breed variable effect was coincident with that of sex for “Brave” breed since only cows and bulls were tested for that breed). The proportion of false-positive reactions decreased with the age expressed in years (OR = 0.87, 95% CI: 0.78–0.96, *p* = 0.006) and was higher in bulls (OR = 2.69, 95% CI: 1.52–4.74, *p* = 0.001) than cows.

## Discussion

In France, gamma-IFN assay is now regularly used either as a serial test to the skin test where its use is to enhance overall disease detection specificity for screening programs in low prevalence areas (13) or in parallel with the intradermal test in order to increase overall disease detection sensitivity in infected herds (16). This second option was chosen in the Camargue region as the prevalence was high. The IFN assay is now considered to be at least as sensitive as the skin test, but its performance is dependent on a large number of factors, including the antigens used, the treatment of samples, the cutoff values used (17) and the interlaboratory reliability (18). The main useful characteristic is that it discloses infection early stages of infection, as early as 14 days post infection (19) but also animals infected with low doses of *M. bovis* (20). One major drawback of the technique could be a lower specificity (21) which can be partially overcome by using recombinant antigens such as ESAT-6 or CFP10 (22) albeit at the cost of reduced sensitivity (23). Since the specificity evaluated with tuberculins was high enough in the Camargue bullfighting cattle population, we decided not to use these antigens in contrast with what is done in other regions in France and elsewhere.

The sensitivity at the individual level seems very low compared to other studies (4, 5, 13, 24). This could be related to the gamma-IFN production failure by bullfight cattle stimulated blood cells compared to other breeds. Moreover, most of the herds were presumed infected for a long time and might contain some anergic animals, which are difficult to detect with *ante-mortem* tests. However, comparisons between studies must be careful as variations such as different cutoff values, the number of animals, the populations, and sample treatment occur between them. Indeed, to obtain accurate sensitivity estimates for a test, all included animals should be slaughtered and tested for confirming infection, whether or not they react to the evaluated test. This is why we only selected data obtained from completely depopulated herds for this study. Furthermore, some meta-analysis estimated a sensitivity of 67% (5, 25) which is a value situated within the CI determined for the sensitivity of the test in bullfights. Finally, even if the individual sensitivity of IFN assay was not optimal, it especially improved the detection at the herd level, because the within-herd prevalence was high (assuming that an average number of three infected animals were present in bTB infected herd). The individual sensitivity of IFN assay remains also much higher than that of the SICT, which is very low in the population of bullfight cattle herds, evaluated around 10%, most probably because of the difficult logistics of administering this test on wild animals. Actually the sensitivity of detection increased more than 30% by using IFN assay in parallel with the SICT (7). As a result of the low sensitivity of SICT in the Camargue region conditions, we observed very low SICT positive rates during the two screening campaigns. Due to the improvement of the epidemiological situation Camargue, a decrease in the proportion of chronically infected animals can be expected. It is therefore possible that the sensitivity of the tests will be higher in this context. However, this lack of individual sensitivity remains an obstacle for the effectiveness of partial cull. This could be counterbalanced by using the IFN assay in parallel with serology, although the first results obtained in Camargue with this latter test have been rather disappointing (26).

No relationship has been observed between (OD PPDB − OD PPDA) and the level of VLs on key organs (e.g., respiratory) of infected animals and thus of the more or less important excretory status of the animals and their risk of infecting other animals in the herd.

Surprisingly, the specificity of IFN assay in this population is higher in comparison with values obtained in other studies (4). These good results allowed a wide approval of the strategy by farmers at early stages of the use of the IFN assay in the field. This high specificity could partly be due to the low production of gamma-IFN by T-cells of this type of animals, but also to the lower exposure of cattle to non-tuberculous mycobacteria in the quite dry environment at the period when animals are sampled for screening (mostly from September to November). Indeed, the proportion of IFN assay false-positive reactions seems closely related to farming area (16, 27). We also observed a higher frequency of non-specific reactions in young animals, which contrasts with results from Gormley et al. (27). Additionally, in Northern Ireland Lahuerta-Marin et al. (16) found increasing risk of false negatives with age. These results could be explained by the presence of higher proportion of natural killer cells in the peripheral blood from young animals (<18 months), which can be a source of innate gamma-IFN production (28). With regards sex, differences of husbandry conditions between males and females could explain the observed more frequent nonspecific results in males. Indeed males’ pastures, which are usually of better quality than those used for females, may be situated in more humid zones where non tuberculous mycobacteria leading to these non-specific reactions are frequent (29). An intriguing question for farmers is the significance of non-VL reactors, which gives the impression that the test has a low specificity and makes its results less credible. This impression was somewhat attenuated by the demonstration of confirmed infected animals (using bacterial culture or PCR) without showing lesions at the slaughterhouse (unpublished data) and results from other studies showing the increased bTB risk when keeping an IFN assay positive animal in a herd from an infected area (6, 8).

The analysis of the ROC curve allowed adapting the characteristics of the assay to field situations and to implement an evolutive control strategy. This work was made easier by simplifying the interpretation criteria, using the S/P ratio which takes into account the interplate absorbance variations and allows a better definition of the required level of detectability for the method. However, the decision area is narrow due to numeric values which are very close to the cut off, for making the difference between infected and non-infected populations. This could be compensated by the use of a method with a higher detectability for a better discrimination of the two populations and maybe a compromise between sensitivity and specificity.

The effect of environmental factors on gamma-IFN production and in particular the breed has already been studied by Schiller et al. (9) who showed that blood sample response to PWM was significantly lower in bullfight herds than classical bovine productions. This could be explained by the influence of stress in this particularly wild breed which is rarely manipulated and selected according to its aggressive behavior for entertainment. This may also explain the lower response to tuberculins more generally during testing (including skin testing) in this population. One of the differences might also be differing levels of T cells between bullfighting and conventional cattle but this has not been shown in this study.

While bovine tuberculosis had been enzootic for several years in bullfighting cattle herds, annual incidence dropped below 1% in 2014 after having regularly risen to above 5% since 2005, with a peak at 10% in 2010. Moreover, all new outbreak detections are now carried by *ante-mortem* testing whereas before the use of the IFN assay 80% of bTB cases were discovered at the slaughterhouse (7). This demonstrates that IFN assay testing on infected herds, in parallel with SICT, is a valuable tool in a bTB eradication program, as already observed by Sinclair et al. (30) and Lahuerta-Marin et al. (16). This was due to the good IFN assay performance in this population but also by facilitating factors in the specific context of the Camargue region: low number of herds, circumscribed geographical area, little exchange with other areas (except few animals from Spain), possibly few environmental non-tuberculous mycobacteria and good communication between stakeholders. Indeed, the acceptability of the measures implemented for animal disease control programs (and particularly for bTB) has an influence on their performance, underlining the importance of a participative approach for their success (31). In contrast, some factors may have limited the speed and effectiveness of sanitation: presence of eventual anergic animals, large size herds, keeping old animals in the herd, poor husbandry practices. The possibility of a wildlife reservoir has also been investigated albeit without demonstrating any case until today, although it appears to be a problem for boars and badgers in other parts of France (32). None of the possible wildlife vector species sampled around cattle herds have been found infected (33), however, the infection pressure was sometimes very high in intensively infected herds as demonstrated by the finding of a case of bTB in a horse sharing pastures in close contact to cattle (34).

Global screening will be continued for 5 more years with two approaches. The first one is a random approach (1/5 of the herds required each year) to demonstrate that the level of prevalence is below 1% (assuming a herd detection sensitivity of 90% and a specificity of 100% by combining the *ante* and *post-mortem* diagnostics). It will be expected that no herd is detected infected among the 200 herds randomly selected every year. The second one, a targeted approach, will increase the chances of detecting the last outbreaks or relapse of infection in herds at risk. The targeted herds will be determined according to the following criteria: former outbreaks (remediated by partial cull), epidemiological link to an outbreak, missing or partial screening, limited abattoir monitoring. Moreover, pre-movement controls will remain mandatory with SICT and IFN assay for all herds.

## Conclusion

The use of gamma-IFN assay in parallel with the single intradermal cervical skin test in a highly infected geographical zone is a valuable tool for a bTB eradication program provided that a good communication between committed stakeholders exists and that the employed screening tests are adapted to the local epidemiological context. In our particular case, the eradication program was also eased by the high specificity of the test in this particular epidemiological context as in other places one of the major issues with IFN assay is the lower specificity, which can have a significant impact when all animals are tested annually. Tuberculosis is now considered under control in the Camargue and Brave races in the Camargue region, particularly through improved early detection in the field. After 10 years of struggle, stakeholders are mobilized and a surveillance system has been designed for the next five years, the objective being to ensure a level of prevalence below 1% before considering progressively lowering of the screening pressure.

## Ethics Statement

The domestic animals used in this study met the definition of “farm animals,” which are not currently covered by French regulations (Décret 2013-118 dated the February 1, 2013, from the French Ministry of Agriculture). The owners of the animals were informed of tests performed on their animals, since all samples were collected during compulsory sanitary investigations.

## Author Contributions

NK conducted the study, was responsible for laboratory work, analyzed data, and drafted the manuscript. M-LB analyzed data and drafted the manuscript. FS and VV participated to the conception of the surveillance program and assisted with data collection. J-LM assisted with data collection. SD conducted the study, analyzed data, and drafted the manuscript.

## Conflict of Interest Statement

The authors declare that the research was conducted in the absence of any commercial or financial relationships that could be construed as a potential conflict of interest.

## References

[1] Cousins DV. *Mycobacterium bovis* infection and control in domestic livestock. Rev Sci Tech (2001) 20:71–85. 10.20506/rst.20.1.1263 11288521

[2] Schiller I RayWaters W Vordermeier HM Jemmi T Welsh M Keck N Bovine tuberculosis in Europe from the perspective of an officially tuberculosis free country: trade, surveillance and diagnostics. Vet Microbiol (2011) 151:153–9. 10.1016/j.vetmic.2011.02.039 21439740

[3] Good M Duignan A. Perspectives on the history of bovine TB and the role of tuberculin in bovine TB eradication. Vet Med Int (2011) 2011:410470. 10.4061/2011/410470 21547209 PMC3087418

[4] De la Rua-Domenech R Goodchild AT Vordermeier HM Hewinson RG Christiansen KH Clifton-Hadley RS *Ante mortem* diagnosis of tuberculosis in cattle: a review of the tuberculin tests, γ-interferon assay and other ancillary diagnostic techniques. Res Vet Sci (2006) 81:190–210. 10.1016/j.rvsc.2005.11.005 16513150

[5] Nuñez-Garcia J Downs SH Parry JE Abernethy DA Broughan JM Cameron AR Meta-analyses of the sensitivity and specificity of ante-mortem and post-mortem diagnostic tests for bovine tuberculosis in the UK and Ireland. Prev Vet Med (2017). 10.1016/j.prevetmed.2017.02.017 28347519

[6] Lahuerta-Marin A Gallagher M McBride S Skuce R Menzies F McNair J Should they stay, or should they go? Relative future risk of bovine tuberculosis for interferon-gamma test-positive cattle left on farms. Vet Res (2015) 46:90. 10.1186/s13567-015-0242-8 26338808 PMC4559371

[7] Keck N Tuberculose bovine en Camargue: apports du test interféron gamma. Point Vet (2010) 309:54–7.

[8] Gormley E Doyle MB Fitzsimons T McGill K Collins JD Diagnosis of *Mycobacterium bovis* infection in cattle by use of the gamma-interferon (Bovigam^®^) assay. Vet Microbiol (2006) 112:171–9. 10.1016/j.vetmic.2005.11.029 16321478

[9] Schiller I Waters WR Vordermeier HM Nonnecke B Welsh M Keck N Optimization of a whole blood interferon-γ assay for detection of *Mycobacterium bovis*-infected cattle. Clin Vaccine Immunol (2009) 16:1196–202. 10.1128/CVI.00150-09 19571108 PMC2725547

[10] Courcoul A Moyen JL Brugère L Faye S Hénault S Gares H Estimation of sensitivity and specificity of bacteriology, histopathology and PCR for the confirmatory diagnosis of bovine tuberculosis using latent class analysis. PLoS One (2014) 9:e90334. 10.1371/journal.pone.0090334 24625670 PMC3953111

[11] Zhang J Abadia E Refregier G Tafaj S Boschiroli ML Guillard B *Mycobacterium tuberculosis* complex CRISPR genotyping: improving efficiency, throughput and discriminative power of ‘spoligotyping’ with new spacers and a microbead-based hybridization assay. J Med Microbiol (2010) 59:285–94. 10.1099/jmm.0.016949-0 19959631

[12] Coad M Downs SH Durr PA Clifton-Hadley RS Hewinson RG Vordermeier HM Blood-based assays to detect *Mycobacterium bovis*-infected cattle missed by tuberculin skin testing. Vet Rec (2008) 162:382–4. 10.1136/vr.162.12.382 18359932

[13] Faye S Moyen JL Gares H Benet JJ Garin-Bastuji B Boschiroli ML. Determination of decisional cut-off values for the optimal diagnosis of bovine tuberculosis with a modified IFNγ assay (Bovigam^®^) in a low prevalence area in France. Vet Microbiol (2011) 151:60–7. 10.1016/j.vetmic.2011.02.026 21420258

[14] Swets JA. Measuring the accuracy of diagnostic systems. Science (1988) 240:1285–93. 10.1126/science.3287615 3287615

[15] Wright PF Nilsson E Van Rooij EM Lelenta M Jeggo MH. Standardisation and validation of enzyme-linked immunosorbent assay techniques for the detection of antibody in infectious disease diagnosis. Rev Sci Tech (1993) 12(2):435–50. 10.20506/rst.12.2.691 8400384

[16] Lahuerta-Marin A McNair J Skuce R McBride S Allen M Strain SA Risk factors for failure to detect bovine tuberculosis in cattle from infected herds across Northern Ireland (2004–2010). Res Vet Sci (2016) 107:233–9. 10.1016/j.rvsc.2016.06.014 27474001

[17] European Food Safety Authority. Scientific Opinion on the Use of a Gamma Interferon Test for the Diagnosis of Bovine Tuberculosis. (2012). Available from: https://www.efsa.europa.eu/fr/efsajournal/pub/2975

[18] Pucken VB Knubben-Schweizer G Döpfer D Groll A Hafner-Marx A Hörmansdorfer S Evaluating diagnostic tests for bovine tuberculosis in the southern part of Germany: a latent class analysis. PLoS One (2017) 12(6):e0179847. 10.1371/journal.pone.0179847 28640908 PMC5481003

[19] Pollock JM Welsh MD McNair J. Immune responses in bovine tuberculosis: towards new strategies for the diagnosis and control of disease. Vet Immunol Immunopathol (2005) 108:37–43. 10.1016/j.vetimm.2005.08.012 16150494

[20] Dean GS Rhodes SG Coad M Whelan AO Cockle PJ Clifford DJ Minimum infective dose of *Mycobacterium bovis* in cattle. Infect Immun (2005) 73:6467–71. 10.1128/IAI.73.10.6467-6471.2005 16177318 PMC1230957

[21] Cagiola M Feliziani F Severi G Pasquali P Rutili D Analysis of the possible factors affecting the specificity of the gamma interferon test in tuberculosis-free cattle herds. Clin Diagn Lab Immunol (2004) 11:952–6. 10.1128/CDLI.11.5.952-956.2004 15358658 PMC515264

[22] Van Pinxteren LAH Ravn P Agger EM Pollock J Andersen P. Diagnosis of tuberculosis based on the two specific antigens ESAT-6 and CFP10. Clin Diagn Lab Immunol (2000) 7:155–60. 10702486 10.1128/cdli.7.2.155-160.2000 PMC95842

[23] Strain SA Mc Nair J Mc Dowell SWJ Bovine Tuberculosis: A Review of Diagnostic Tests for M. bovis Infection in Cattle. Belfast: Agri-Food and Biosciences Institute (2011).

[24] Alvarez J Perez A Bezos J Marqués S Grau A Saez JL Evaluation of the sensitivity and specificity of bovine tuberculosis diagnostic tests in naturally infected cattle herds using a Bayesian approach. Vet Microbiol (2012) 155(1):38–43. 10.1016/j.vetmic.2011.07.034 21890284

[25] Downs SH Parry J Nunez-Garcia J Abernethy DA Broughan JM Cameron AR Meta-analysis of diagnostic test performance and modelling of testing strategies for control of bovine tuberculosis. Proc Soc Vet Epidemiol Prev Med (2011) 23:139–53.

[26] Moyen JL Gueneau E Keck N Gares H Boschiroli ML First assessment of the use of serology for the diagnosis of bovine tuberculosis in France. Epidemiol Santé Anim (2014) 65:41–51.

[27] Gormley E Doyle M Duignan A Good M More SJ Clegg TA Identification of risk factors associated with disclosure of false positive bovine tuberculosis reactors using the gamma-interferon (IFNγ) assay. Vet Res (2013) 44:117 10.1186/1297-9716-44-117 24308747 PMC4028746

[28] Olsen I Boysen P Kulberg S Hope JC Jungersen G Storset AK. Bovine NK cells can produce gamma interferon in response to the secreted mycobacterial proteins ESAT-6 and MPP14 but not in response to MPB70. Infect Immun (2005) 73:5628–35. 10.1128/IAI.73.9.5628-5635.2005 16113280 PMC1231097

[29] Biet F Boschiroli ML. Non-tuberculous mycobacterial infections of veterinary relevance. Res Vet Sci (2014) 97:S69–77. 10.1016/j.rvsc.2014.08.007 25256964

[30] Sinclair JA Dawson KL Buddle BM. The effectiveness of parallel gamma-interferon testing in New Zealand’s bovine tuberculosis eradication programme. Prev Vet Med (2016) 127:94–9. 10.1016/j.prevetmed.2016.03.020 27094146

[31] Calba C Goutard F Vanholme L Antoine-Moussiaux N Hendrikx P Saegerman C. The added-value of using participatory approaches to assess the acceptability of surveillance systems: the case of bovine tuberculosis in Belgium. PLoS One (2016) 11:e0159041. 10.1371/journal.pone.0159041 27462705 PMC4962975

[32] Richomme C Boadella M Courcoul A Durand B Drapeau A Corde Y Exposure of wild boar to *Mycobacterium tuberculosis* complex in France since 2000 is consistent with the distribution of bovine tuberculosis outbreaks in cattle. PLoS One (2013) 8(10):e77842. 10.1371/journal.pone.0077842 24167584 PMC3805591

[33] Rivière J Réveillaud E Boschiroli ML Hars J Richomme C Faure E Sylvatub: results of a one year of surveillance of tuberculosis in wildlife in France. Bull Epidemiol Santé Anim Alim (2013) 57:10–5.

[34] Keck N Dutruel H Smyej F Nodet M Boschiroli ML Tuberculosis due to *Mycobacterium bovis* in a Camargue horse. Vet Rec (2010) 166:499–500. 10.1136/vr.b4785 20400743

